# Reference point detection for camera-based fingerprint image based on wavelet transformation

**DOI:** 10.1186/s12938-015-0029-1

**Published:** 2015-04-30

**Authors:** Mohammed S Khalil

**Affiliations:** Faculty of Commerce and Economics, Sana’a University, Sana’a, Yemen; Center of Excellence Information Assurance, King Saud University, Riyadh, Saudi Arabia

**Keywords:** Mobile phone, Camera, Ridge analysis, Wavelet, Core-point detection, Reference point, Fingerprint

## Abstract

**Background:**

Fingerprint recognition systems essentially require core-point detection prior to fingerprint matching. The core-point is used as a reference point to align the fingerprint with a template database. When processing a larger fingerprint database, it is necessary to consider the core-point during feature extraction. Numerous core-point detection methods are available and have been reported in the literature. However, these methods are generally applied to scanner-based images. Hence, this paper attempts to explore the feasibility of applying a core-point detection method to a fingerprint image obtained using a camera phone.

**Method:**

The proposed method utilizes a discrete wavelet transform to extract the ridge information from a color image. The performance of proposed method is evaluated in terms of accuracy and consistency. These two indicators are calculated automatically by comparing the method’s output with the defined core points.

**Results:**

The proposed method is tested on two data sets, controlled and uncontrolled environment, collected from 13 different subjects. In the controlled environment, the proposed method achieved a detection rate 82.98%. In uncontrolled environment, the proposed method yield a detection rate of 78.21%.

**Conclusion:**

The proposed method yields promising results in a collected-image database. Moreover, the proposed method outperformed compare to existing method.

## Introduction

Nowadays, smartphones and tablet PCs are commonly used and are stimulating the utilization of mobile ecommerce, which has significantly increased. This is because a smartphone or tablet PC has a powerful computing capability and is portable and easy to use. As an example, one of the most popular smartphone applications is mobile banking (M-Banking) [1]. M-Banking has become famous because the mobility of a smartphone or table PC makes it possible to conduct any kind of electronic transaction related to banking services at any time and place. Several recent works have reported recognition methods for personal biometrics using a smartphone [2-4]. Although biometric authentication on a smartphone or tablet PC is very challenging, it will provide many benefits in the future.

One of the most popular biometric methods being implemented in the public sector is fingerprint recognition. The uniqueness and immutability of fingerprints with aging have been proven. This encourages the rapid growth of the device technology for fingerprint scanners. In general, such a device has a flat scanner, upon which the tip of a finger is placed. This action allows the scanner lens to easily acquire the ridges and valleys of the fingerprint imprinted on the surface. Further, the acquired fingerprint can be matched with a stored template in a fingerprint collection.

Prior to matching the fingerprint, it is necessary to extract some features from the fingerprint image. Feature extraction for fingerprint recognition can be classified into two types called local-feature and global-feature extractions. Local-feature extraction considers the small details or minutiae of a fingerprint ridge. In contrast, global-feature extraction considers the flow pattern of the whole fingerprint. The literature shows that global features are more robust than local features in a large database [5]. Hence, this paper considers unresolved issues related to global features.

**Figure 1:**
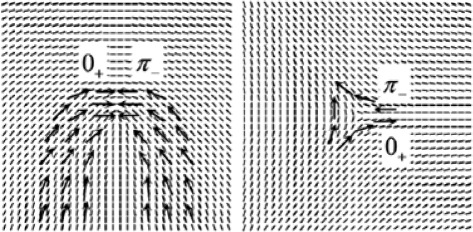
Example of flow ridges found in fingerprint (re-printed from [32]).

**Figure 2:**
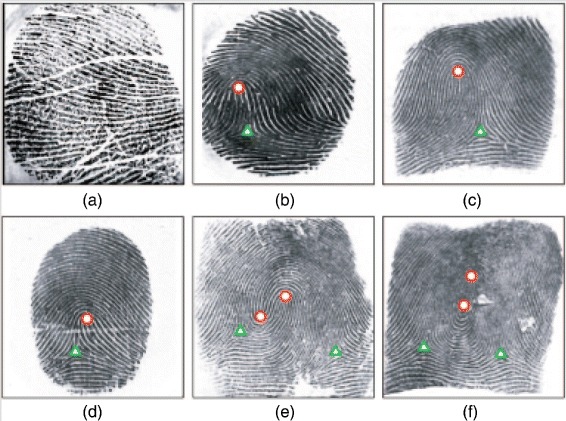
Known six types of fingerprints that acquire using scanner-based along with core-point denoted with circle and delta-point denoted with triangle. The known size types are **(a)** Plain-arch, **(b)** Tented-arch, **(c)** Left-loop, **(d)** Right-loop, **(e)** Twin-loop, **(f)** Whorl (re-printed from [20]).

At the global level, a fingerprint can be presented as a flow of ridges with symmetric properties, as depicted in Figure 1. The orientation flow that drastically changes, as denoted by the thick arrow in Figure 1, is called the core point or singular point. Two unknown fingerprint images can be aligned with reference to the core point. In addition, it can be utilized to classify the fingerprint type [6,7]. Thus, it is very important to properly identify the core point [6-14]. Figure 2 shows the known distinctive types of fingerprints, and the corresponding singular points are marked.

The common problems reported for large-scale databases, including the variety of fingerprint types and poor image quality, can be solved with the help of the reference point [6,15,16]. Currently, core-point detection methods have only been proposed for scanner-based images. Numerous studies, as reported in the literature, have proposed new methods for the analysis and detection of the core points in scanner-based fingerprint images. In general, the existing methods can be classified into two categories. The first method utilizes the Poincare index to locate the core point. This algorithm computes the total orientation variation around a point to determine whether a core point is present. The second method uses template matching or ridge, probability, or shape analysis [8,16-18]. In real applications, Poincare index-based methods have been proven to be more robust than the second method because they are able to handle image rotation. Moreover, even though the computation cost is high, it is still acceptable.

**Figure 3:**
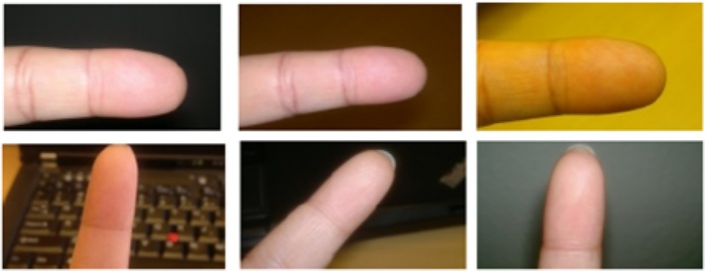
Various fingerprint image acquire using a camera and taking in different angle and environment (re-printed from [33]).

Meanwhile, no study can be found related to the use of core-point detection for camera-based images. The characteristics of a camera-based image are totally different than those of a scanner-based image. As seen in Figure 3, it can be blurred and could have been captured at a different angle or illumination and have other issues. Hence, it is challenging to explore a core-point detection method that can be applied to camera-based images. Prior to discussing the detection method, recent core-point detection methods are discussed to show the state-of-the-art.

## Related works

Poincare’s index (PI) method is frequently used in the early stage of fingerprint recognition systems [6,10,11,19,20]. This conventional method considers the closed curve in order to locate the core point. The Poincare index, along with its properties, can be defined as follows:

- Define *V* as a vector field identified as a continuous two-dimensional vector as follows:
  (1) $$ V(x,y)=p(x,y)+i.q(x,y)  $$
- The Poincare index of *V*(*x*,*y*) within an unsystematic simple bounded path *γ* can be presented as:
  (2) $$ I(\gamma)=\frac{1}{2\pi} \int_{(x,y)\in\gamma} d\phi(x,y)  $$
  (3) $$ \phi(x,y)=arg\ V(x,y)  $$
  where *ϕ*∈[0,2*π*) represents the angle at coordinate (*x*,*y*). The integration is taken contrary to clockwise within *γ*.
- The Poincare index within the predefined region is equivalent to the summation of the singular point Poincare indices inside the predefined region. It can be defined as follows:
  (4) $$ \sum_{k}I(\gamma_{k})=I(\Gamma_{E})-I(\Gamma_{I})  $$
- If a singular point does not exist on two homotopic closed paths, it has the same Poincare indices.

The main drawbacks of this method are its high computation cost, inability to handle a wide range of fingerprint types, and extreme sensitivity to noise [20]. Poincare index-based algorithms typically produce countless unauthentic detections, particularly when dealing with degraded fingerprint images, even after applying image enhancement or post-processing on spurious detections. This issue brings problems in many applications, resulting in poor performance. There are two reasons that such an outcome can occur. First, the feature of the Poincare index itself is not strong enough to accurately detect a singular point. Second, spurious detections should reduce not only based on local characteristics but also global discriminative is necessary to be incorporated. Perona [21] reported an interesting method called orientation diffusion. In the dynamic diffusion process, Perona utilized the global constraint of oriented texture.

The sine-map-based approach [12] processes two defined regions of a fingerprint image and then calculates the sine component. Then, the sine component is analyzed using a multiple resolution analysis to locate the core point. Jain et al. reported that their method is resistant to rotation and noise. However, the sine component needs perfect circulars of the fingerprint to achieve good result.

Liu et al. [15] considered the orientation uniformity in a fingerprint image to trace the core point. This method only considers the orientation field and curvature direction, which is suitable only for limited fingerprint types. Quite similar to the method of Liu et al. was the curvature-based technique introduced by Van and Le [22]. However, their method was found to be weak when handling plain arch ridges and twofold core-point fingerprints.

Numerous features have been reported in the literature, including a pixel-wise orientation field [23,24], orientation curvature [17,18,25], and template model matching [26]. Such methods are known to have the same drawbacks of being unable to handle the degraded fingerprint images that might occur in scanner-based images and even more frequently in camera-based images.

Recently, Le and Van [5] proposed a core-point detection method that employs vertical variation and rotational symmetry features. An experiment was performed on a standard database called FVC2004 DB1. The result showed that this method was able to handle the fingerprint variety in the dataset. Then, Bahgat et al. [27] utilized an orientation map that was presented in grayscale to locate the core point. They assumed that the core point should appear at the end of the discontinuous line in the orientation map. In addition, a predefined equation was used to verify the core point. Their method was tested on the FVC2002 and FVC2004 datasets, and their reported results were better than those of the fast edge-map based method [28].

A comprehensive survey by Khalil et al. [29] on core-point detection reported that the literature contains some initial studies related to camera-based images. However, there are many gaps still to be resolved regarding the implementation of a robust core-point detection method for camera-based images. Hence, this paper proposes a method for locating the core point in a fingerprint image acquired using a camera.

## Method

**Figure 4:**
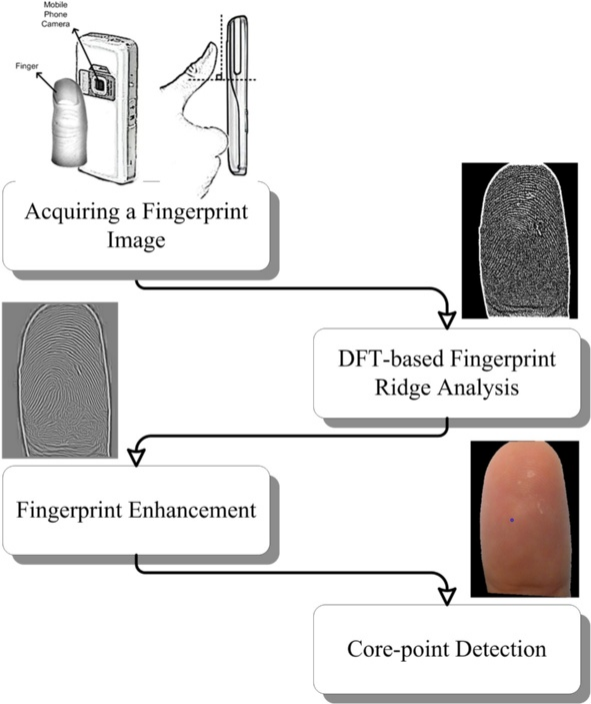
General overview of the proposed method.

A general overview of the proposed method is given in Figure 4, and explanations are provided in the next sub-sections.

### DFT-based fingerprint ridge analysis

**Figure 5:**
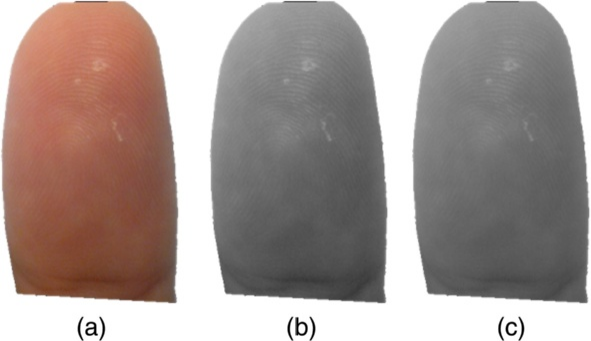
RGB **(a)** to grayscale conversion **(b)** and the normalized image **(c)**.

Following fingerprint image acquisition using a camera, the fingerprint image in the red, green, and blue format is converted to the grayscale format to reduce the illumination problem due to the use of several lighting sources. Such conversion is important because the lighting issue has been recognized as one of the foremost aspects that degrade the detection performance. In addition, fingerprint enhancement using STFT analysis (see section ‘Fingerprint enhancement’) requires a normalized image to obtain good results. The simple normalization algorithm [30] computes the variance and mean of the image to reduce the illumination variation. Using equation 5 below, a normalized image is produced:

(5) $$ g(x,y)=\frac{f(x,y)-m_{f}(x,y)}{\sigma_{f}(x,y)}  $$

where *f*(*x*,*y*) defines the original fingerprint image, *m*_*f*_(*x*,*y*) is the calculated estimation of the mean of *f*, and *σ*_*f*_(*x*,*y*) presents the assessment of the standard deviation from the original image. The assessments for *m*_*f*_ and *σ*_*f*_ are produced from spatial smoothing. Figure 5 shows the original fingerprint image converted into the grayscale format and the final normalized image.

**Figure 6:**
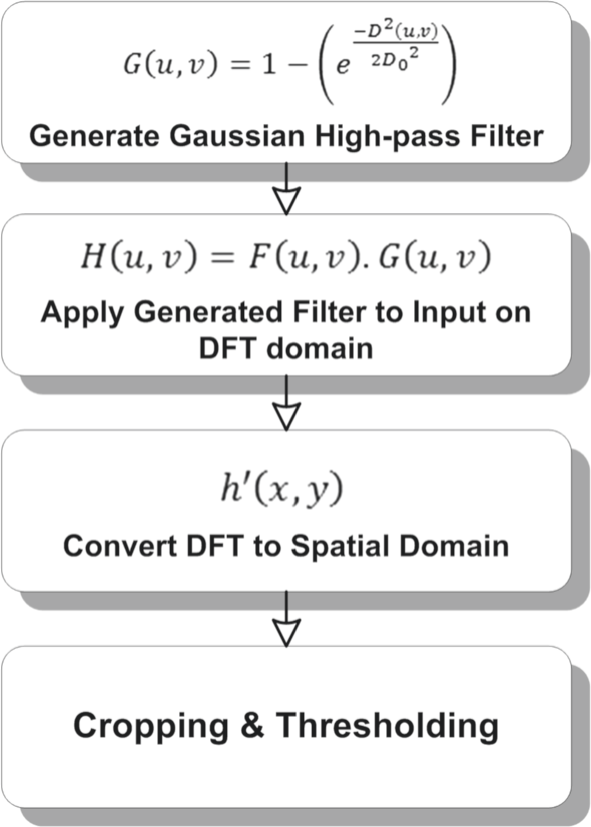
Overview of ridge analysis using DFT.

The surface of a fingertip contains a unique pattern formed by ridges and valleys, which is known as the fingerprint. Both the ridges and valleys create a circular shape as they repeatedly run in parallel and sometime diverge. The pattern can be revealed through a ridge analysis method, as presented in this paper. A high-pass filter is applied to the input image through a discrete Fourier transform (DFT) to reflect the ridge information. The ridge information is used later to locate the core point. The proposed DFT-based ridge analysis for a fingerprint image is presented in Figure 6.

In the first step, a Gaussian high-pass filter, *G*(*u*,*v*), is created by taking a padding size (*PxQ*) and a filter width for the Fourier transform process as defined in equations 6, 7, and 8.

(6) $$ D_{0}=0.05 \times P  $$

(7) $$ D(u,v)=\sqrt{\left(u-\frac{P}{2} \right)^{2} + \left(v-\frac{Q}{2} \right)^{2}}  $$

(8) $$ G(u,v)=1-\left(e^{\frac{-D^{2}(u,v)}{2{D_{0}}^{2}}} \right)  $$

**Figure 7:**
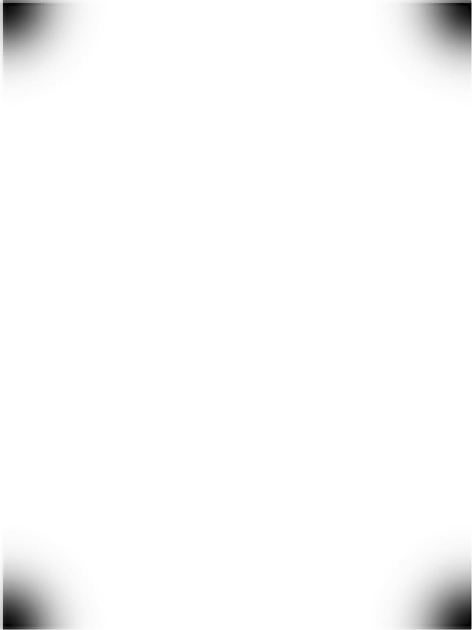
high-pass filter that generated using Gaussian function in DFT domain.

Figure 7 illustrates the Gaussian high-pass filter utilized in the proposed method. The padding size is computed with respect to the input size, 2∗(*M*×*N*). Afterward, a proper filter width is selected during the experiments, where the most suitable filter width is 5% of the padding size, *P*. The input image, *f*(*x*,*y*), is transformed into the wavelet domain using equation 9.

(9) $$ F(u,v)=\sum_{x=0}^{M-1}\sum_{y=0}^{N-1} f(x,y)e^{-i2\pi\left(\frac{ux}{M}+\frac{vy}{N}\right)}  $$

**Figure 8:**
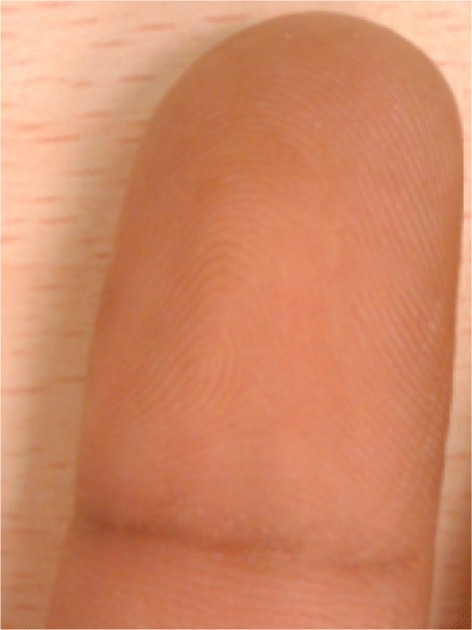
Fingerprint image acquired using mobile phone camera.

**Figure 9:**
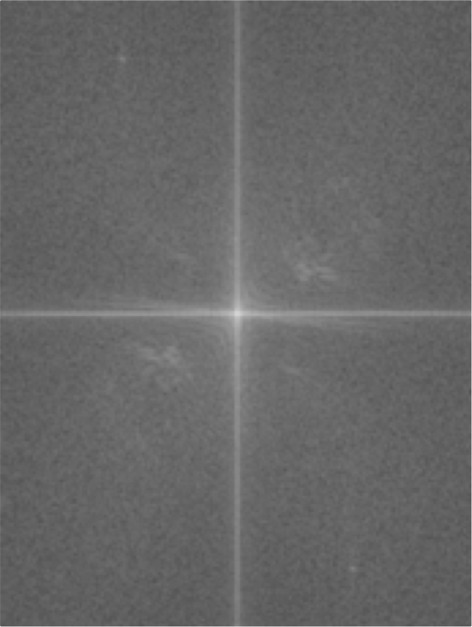
Fingerprint image (Figure 8) in Fourier domain.

**Figure 10:**
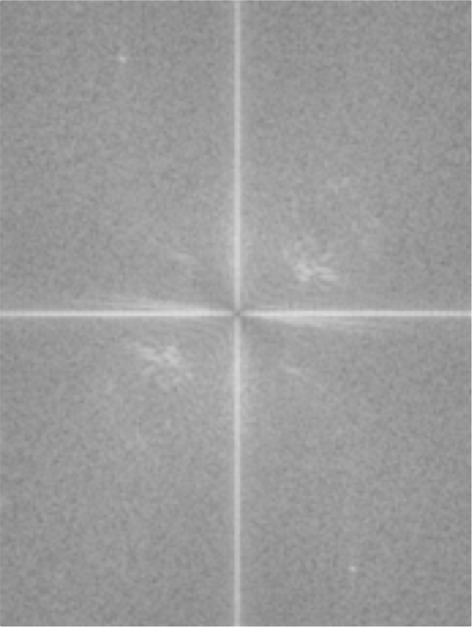
Output of scalar product between input image and the filter.

**Figure 11:**
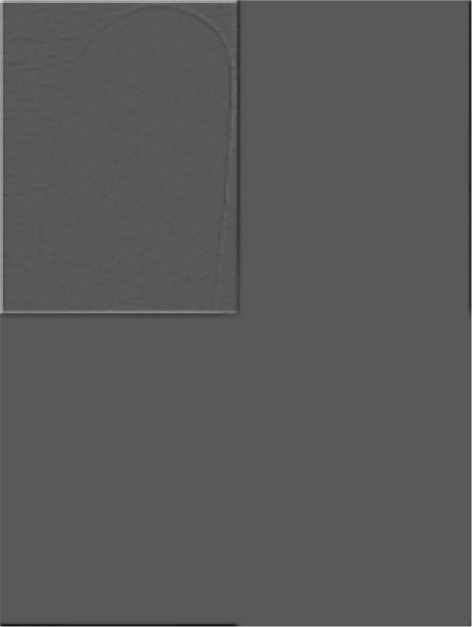
Output of scalar product (Figure 10) in spatial domain.

Figures 8 and 9 present the input and result of the conversion process, respectively. The filter, *G*(*u*,*v*), is employed as a scalar product of the matrices of the input image, *F*(*u*,*v*), in the Fourier domain using equation 10, as drawn in Figure 10. Then, the matrix *H*(*u*,*v*) is reverted back into the spatial domain using the inverse function in equation 11. The result is depicted in Figure 11.

(10) $$ H(u,v)=F(u,v).G(u,v)  $$

(11) $$ h'(x,y)=\frac{1}{MN} \sum_{u=0}^{M-1}\sum_{v=0}^{N-1} H(u,v)e^{-i2\pi\left(\frac{ux}{M}+\frac{vy}{N}\right)}  $$

**Figure 12:**
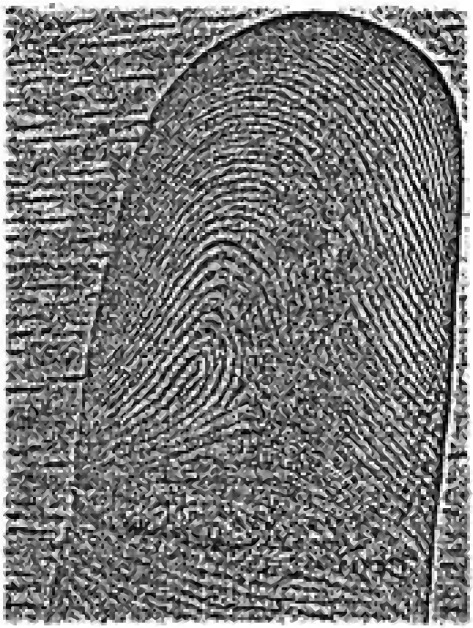
Output of proposed ridge analysis.

In the last step, the generated image is cropped to eliminate the preceding padding. The ridge information is obtained based on a simple threshold, as defined in Equations 12 and 13. Figure 12 presents the result of the DFT-based fingerprint ridge analysis.

(12) $$ I_{ridge}=\left\{ \left.I_{ridge}(i,j) \in h' \right| i=1,\ldots,I_{h},j=1,\ldots,I_{w} \right\}  $$

(13) $$ I_{ridge}= \left\{\begin{array}{ll} 1,& I_{ridge} (i,j)>0.005 \times max (I_{ridge})\\ 0,& \text{otherwise} \end{array}\right.  $$

### Fingerprint enhancement

**Figure 13:**
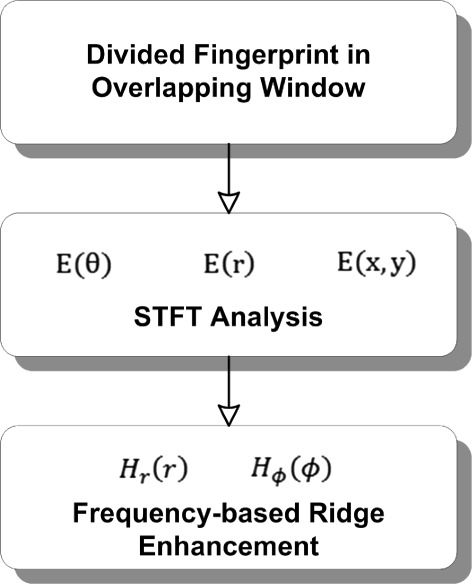
Overview of fingerprint enhancement.

Locating the core point and extracting the fingerprint features are critical steps. Therefore, a fingerprint improvement is preferred to attain a high-quality image for the fingerprint verification step. In this paper, the short time Fourier transform (STFT) approach is utilized to enhance the fingerprint image [31]. Figure 13 depicts the fingerprint enhancement diagram, which consists of two major steps. Initially, the image of the ridge information is split into overlapping windows. These overlapping windows are considered to maintain the continuity of the ridges and eliminate the block effect that naturally occurs as a result of block-by-block operations. The small window preserves the invariance of a small region of the image and is easily modeled as a surface wave. In every window, the STFT analysis is applied to produce a ridge orientation image (ROI), energy image (EI), and ridge frequency image (RFI). Prior to performing the STFT analysis, the Fourier spectrum of the window *F*(*r*,*θ*) is first presented in a polar system. Then, two functions called the marginal density function {*p*(*θ*),*p*(*r*)} and probability density function *p*(*r*,*θ*) are used to generate the ROI, as calculated below:

(14) $$ p(r,\theta)=\frac{\left|F(r,\theta)\right|^{2}}{\int_{r}{\int{\theta{\left|F(r,\theta)\right|^{2}}}}}  $$

(15) $$ p(r)=\int_{\theta}{p(r,\theta)d\theta}  $$

(16) $$ p(\theta)=\int_{r}{p(r,\theta)dr}  $$

The orientation value denoted as *θ* is a variable that is understood to be a random value, and it has the probability density function denoted by *p*(*θ*). Using equation 17, the ROI value shown below can be obtained. Afterward, vector averaging is applied to smooth the orientation image.

(17) $$ E(\theta)=\frac{1}{2} tan^{-1} \left(\frac{\int_{\theta}{p(r,\theta)d\theta}}{\int_{r}{p(r,\theta)dr}} \right)  $$

Similar to the above, the ridge frequency *r* is presumed to consist of random values, along with function *p*(*r*), which is known as the function of the probability density. RFI is calculated using equation 18 below, and it is smoothed by applying 3×3 Gaussian mask to it.

(18) $$ E(r)=\int_{r} {p(r).rdr}  $$

Afterward, a coherence image is generated from the smoothed orientation image. This coherence image is required to prevent spurious artifacts. Such artifacts occur because the ridge flow stops at the block edge, particularly at high curvature regions next to the core points. It is known that around the core point, numerous dominant orientations are present.

Prior to refining the fingerprint image, the energy image is computed using Equation 19, as shown below. The Otsu’s well-known thresholding is utilized to obtain the region mask.

(19) $$ E(x,y)=log\left\{ \int_{r}{\int_{\theta}{\left|F(r,\theta)\right|^{2}}} \right\}  $$

**Figure 14:**
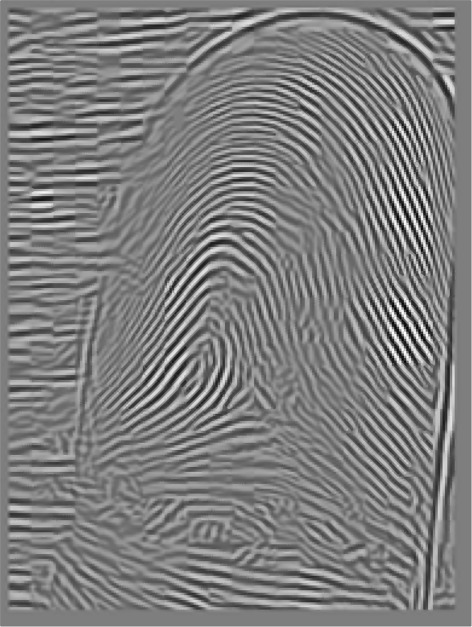
Final image after fingerprint enhancement.

Two filters called a radial filter (Equation 20) and angular filter (Equation 21) are obtained from the ridge orientation matrix, coherence matrix, and frequency matrix. These filters are utilized to smooth the image. The filter is applied on overlapping 16×16 blocks in the Fourier spectrum [31]. These two filters are defined below:

(20) $$ H_{r}(r)=\sqrt{\frac{\left(rr_{BW}\right)^{2n}}{\left(rr_{BW}\right)^{2n}+\left(r^{2}+r_{BW}^{2}\right)^{2n}}}  $$

(21) $$ H_{\phi} (\phi)= \left\{\begin{array}{ll} \frac{cos^{2}\pi\left(\phi-\phi_{c}\right)}{2\phi_{BW}},& \left|\phi-\phi_{c}\right| \leq \phi_{BW} \\ 0, & \text{otherwise} \end{array}\right.  $$

where *r*_*BW*_ is the radial bandwidth, *ϕ*_*BW*_ is the angular bandwidth, and *ϕ*_*c*_ is the mean orientation. Finally, after applying the above-mentioned filters, the Fourier spectrum is reconstructed back into the spatial domain. Figure 14 depicts the fingerprint image following the enhancement step.

### Locating the core-point

**Figure 15:**
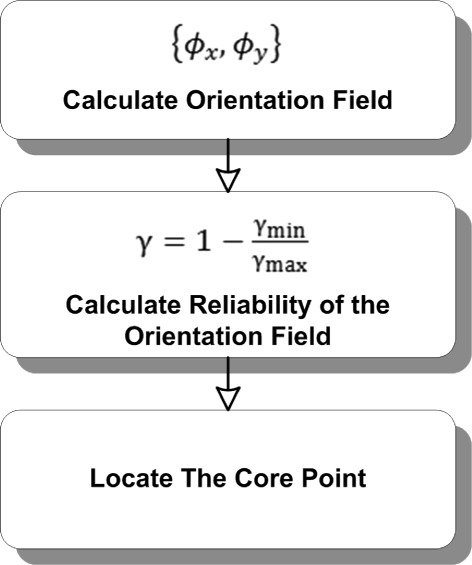
Overview of core-point detection method.

Looking at the convex ridge information, the core point can be defined as the point that has the highest curvature. One way to locate the dominant orientation is by considering the reliability of the orientation field. The reliability of the orientation field provides flexibility to analyze the dominant orientation for various types of fingerprints. Figure 15 presents a diagram of the proposed method to locate the core point.

The preliminary step to discovering the core point is computing the orientation field. Therefore, the fingerprint image is split into non-overlapping blocks, with the block size fixed at 16×16. Then, each block is defined by a single orientation that matches the foremost orientation. However, this orientation might not be accurate because of various issues, including degraded valley and ridge structures, noise, and low contrast. Therefore, a low-pass filter is utilized on the local ridges to smooth the flawed orientation. Finally, the orientation of the ridges is calculated based on the vertical *G*_*yy*_ and horizontal *G*_*xx*_ gradients and the orientation image {*ϕ*_*x*_,*ϕ*_*y*_}, denoted as follows:

(22) $$ G_{xx}=\sum_{(x,y)\in w}{{G_{x}^{2}} (x,y)}  $$

(23) $$ G_{yy}=\sum_{(x,y)\in w}{{G_{y}^{2}} (x,y)}  $$

(24) $$ G_{xy}=\sum_{(x,y)\in w}{G_{x} (x,y). G_{y} (x,y)}  $$

(25) $$ \theta(x,y)=\frac{1}{2} \tan^{-1} \frac{2G_{xy}}{G_{xx}-G_{yy}}  $$

(26) $$ \phi_{x}=\cos(2\theta(x,y))  $$

(27) $$ \phi_{y}=\sin(2\theta(x,y))  $$

Subsequently, the maximum reliability peak is computed to obtain the greatest curvature based on vectors of the gradients *G* and filtered orientation *ϕ*^′^ using the equation shown below:

(28) $$ \gamma_{min}=\frac{\left(\left(G_{xx}+G_{yy}\right)-\left(\phi_{x}' G_{xx}+G_{yy}\right)-\left(\phi_{y}' G_{xy} \right) \right)}{2}  $$

(29) $$ \gamma_{max}=G_{xx}+G_{yy}-\gamma_{min}  $$

(30) $$ \gamma=1-\frac{\gamma_{min}}{\gamma_{max}}  $$

**Figure 16:**
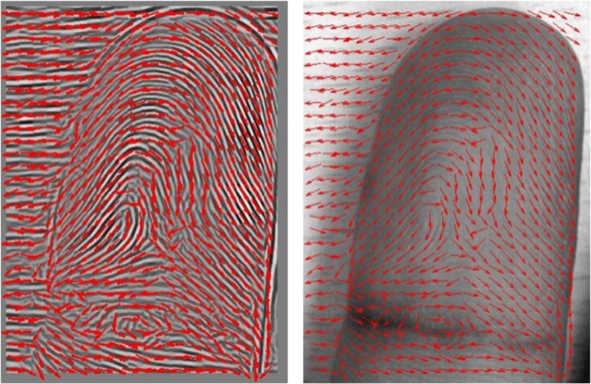
Enhanced ridge image (left) and input image (right) overlapped with orientation Field.

**Figure 17:**
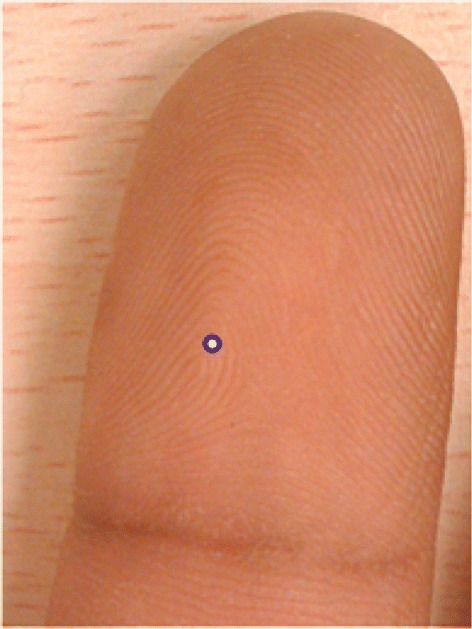
Locating core point.

The result of applying Equation 27 for generating the orientation field is presented in Figure 16. The orientation {*ϕ*_*x*_,*ϕ*_*y*_} is shown by a short arrow in Figure 16 and points at a different angle. Afterward, equation 30 is computed to obtain the reliability peak prior to locating the core point. There are three main steps to locate the core point. Initially, two distinct regions are decomposed from the orientation field reliability by considering the predefined range of values. Afterward, a thinning operation is applied to obtain the vital connectivity and contour of the original orientation profile. The core point should appear on a ring-shaped contour. Hence, non-ring-shaped contours are removed using a shrinking operation. The final core point is obtained by applying a shrink and fill operation. The core-point location of the input image in Figure 8 is depicted in Figure 17.

## Experimental results

The image input used in this paper is acquired by utilizing the camera on a mobile device, HTC magic (3.5 megapixels). Fingerprint images are acquired from 13 subjects. There are two scenarios in our dataset. In the first scenario, the environment was controlled, and the fingerprints were captured on a white background. The dataset for the 1st scenario is called dataset A, which consists of 94 fingerprint images. In the other scenario, the images were acquired in a natural way without any human intervention. These images are called dataset B, which consists of 546 fingerprint images. This dataset is known to have unsteady angles due to the natural acquisition, uncontrolled environment lighting, and blur issue. The unsteady angle occurred because the fingerprint and camera were not placed at fixed positions. The uncontrolled environment lighting created illumination issues, which are challenging for core-point detection. The blur issue occurred when the camera was unable to focus on the fingerprint or the fingerprint itself was not stable.

**Figure 18:**
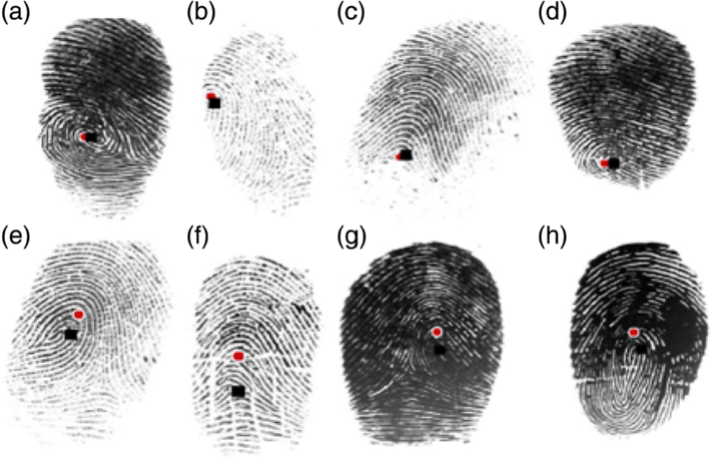
Le & Van [5] evaluated their method with reference points that detected by human experts (denoted with black square).**(a)**–**(d)** example of their accepted cases where the detected core-point has distance error is less than 20 pixels, and **(e)**–**(h)** shows their false cases, which is the detected core-point has distance error greater than 20 pixels. (this figure re-printed from [5]).

The performance of the proposed method is evaluated in a way similar to Le & Van’s experiments [5], as presented in Figure 18. An attempt is made to evaluate the consistency and accuracy. In this regard, a fingerprint expert manually assigned the core point on each fingerprint image. These data are used as references for the detected core points generated by the proposed method. The Euclidean distance between each core point assigned by the expert and detected by the proposed method is calculated. The result is considered to be the distance error from the predefined core point. Similar to Ref. [5,15], the acceptable distance error should be less than 20 pixels. According to the literature, a distance error greater than 20 pixels is a significant error and counted as a false detection. This is because such a detected core point can be classified as a false core point, which drastically affects the subsequent processing steps. A smaller value for the distance error means better accuracy. The detection rate is counted from the number of correctly detected core points with a distance error of less than 20 pixels. The false alarm rate is identified as the wrongly identified core points with a distance error greater than 40 pixels.

**Figure 19:**
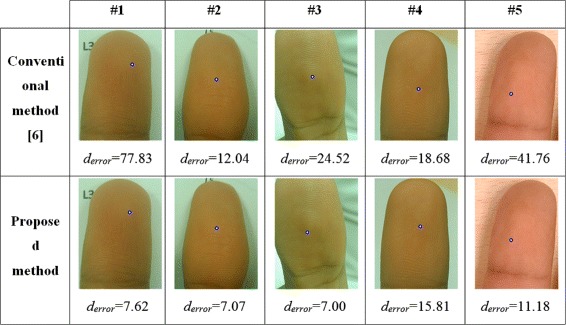
Some comparison results of singular point detection. First row is the detected results using conventional algorithm [6] and second row is using the proposed algorithm.

The experiment shows that the proposed method is able to appropriately identify the core points in the dataset. The method accuracy is assessed based on the distance error as mentioned above. The proposed method achieves a detection accuracy of up to 82.98% on dataset A and 78.21% on dataset B. Meanwhile, the conventional method mostly fails to locate the core points in both dataset A and dataset B. The conventional method can achieves an accuracy of less than 55%. Tables 1 and 2 present comparisons between the proposed method and conventional method. Figure 19 depicts some results for the detected core points, denoted by small circles, that were obtained using the proposed approach and conventional method.

**Table 1:** **The comparison result of proposed method and conventional method on dataset A**

	**Rates (%)**	**Proposed method**	**Conventional method [** 6 **]**
**Core-point**	Detection rate	82.98	46.81
	False Alarm Rate (FAR)	4.62	48.94

**Table 2:** **The comparison result of proposed method and conventional method on dataset B**

	**Rates (%)**	**Proposed method**	**Conventional method [** 6 **]**
**Core-point**	Detection rate	78.21	53.85
	False Alarm Rate (FAR)	6.78	36.81

**Figure 20:**
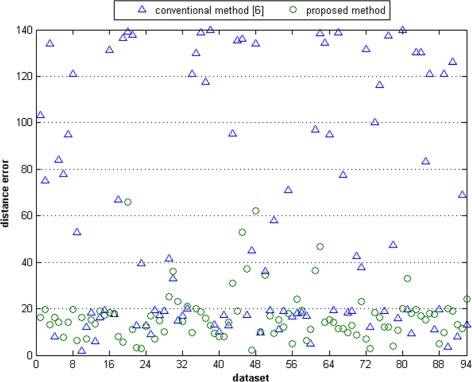
The distance error result on dataset A.

**Figure 21:**
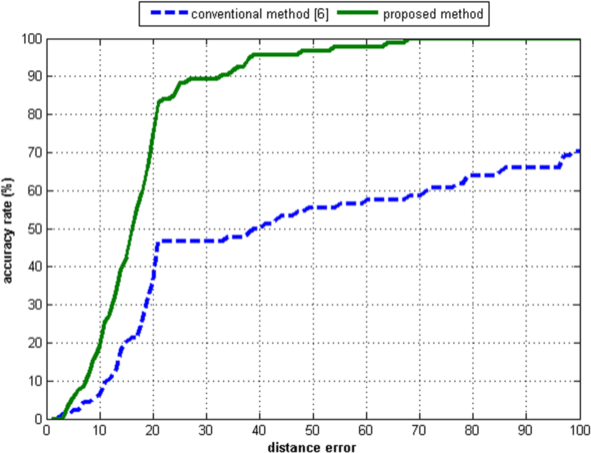
The accuracy rate result on dataset A.

**Figure 22:**
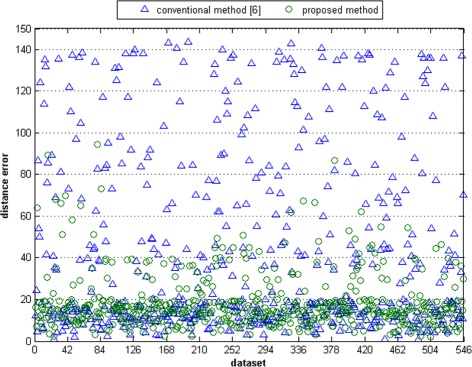
The distance error result on dataset B.

**Figure 23:**
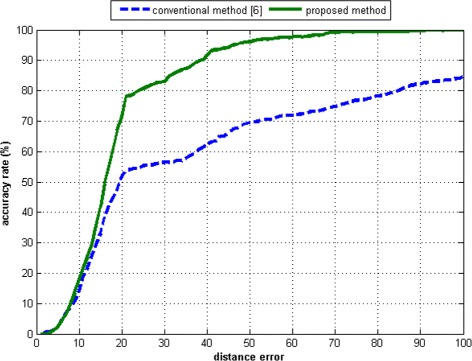
The accuracy rate result on dataset B.

Figures 20, 21, 22, 23 present the distributions of the distance errors and accuracy of the proposed method and conventional method. As seen in Figure 21, the proposed method can reach an accuracy of 90% on dataset A when the distance error considers up to 30 pixels. In the larger dataset B, the proposed method can achieve that same accuracy only if the distance error considered is less than 40 pixels, as depicted in Figure 23. On the other hand, the conventional method is unable to reach such an accuracy even after considering a large distance error of 100 pixels. The experimental results show that the proposed method outperforms the conventional method. This is because the conventional method is actually intended for scanner-based images. The conventional method was mostly unable to detect the core point properly. It was proven that the proposed ridge analysis using the Fourier transform is able to construct clear ridges from a camera-based image. The ridge analysis is critical because the subsequent steps are correlated with the output of the ridge analysis step.

Based on observations, the wrong results occurred as a result of degraded fingerprint images. In this paper, blurry images were typically inescapable because of shaky hands. It is known that if the ridge information becomes fragile, it is hard to reveal the pattern. Therefore, the proposed ridge analysis method was incapable of properly extracting the ridges of a fingerprint. Once an inaccurate ridge is produced, the computed orientation field has the wrong direction, which generates an erroneous core point. Nevertheless, this condition does not often occur and can be disregarded. In addition, the latest cameras included in mobile devices include a built-in image stabilizer to reduce the blur-motion issue.

## Conclusions and future works

The state-of-the-art core-point detection methods for scanner-based fingerprint images have been discussed, and it was found that most of the existing methods are intended specifically for scanner-based images. Recently, there has been promising research that exploits the camera on a mobile phone to capture a fingerprint image, and attempts have been made to authenticate such an image. This recent research motivated this paper to fill the research gap, particularly on core-point detection methods.

The authentication of biometric data such as fingerprints using a mobile phone will increase and open up a wide range of handy applications, including banking, device protection, and other daily tasks that require individual authentication. However, it is too early to release the system to the public, as more studies are needed, especially concerning the analysis of the image quality, image enhancement, pre-processing, feature extraction, and securing the biometric data.

In this paper, a core-point detection method was proposed and tested on two datasets that considered both controlled and uncontrolled environments. A comparison was also carried out with a widely used robust core-point detection method intended for scanner-based images. In the controlled environment, the proposed method outperformed the conventional method with a detection rate of 82.98%. Afterward, in dataset B, where the fingerprint images were taken without controlling the environment, the proposed method again surpassed the conventional method with a detection rate of 78.21%. The proposed method can locate the core point with the allowable distance error of 20 pixels. Hence, ridge analysis using the DFT-based approach has been proven to produce enough information to find the core point.

In addition, the experiment showed that core-point detection methods suffer under an uncontrolled environment. Both methods obtained less detection accuracy on dataset B. This occurred because a fingerprint image taken using a camera in a natural environment usually has motion blurs, under-lighting, or over-lighting, and the camera itself may fail to focus on the object. Therefore, more study to improve the image quality is necessary to solve this issue.

Bringing fingerprint authentication to a mobile phone device has great potential for the future. However, additional studies must be conducted to achieve acceptable accuracy. One of the crucial issues on this topic is degraded input images. Problems such as blurry fingerprints, unstable images due to shaky hands, low-resolution images, and uncontrolled environments that create unsatisfactory lighting requirements affect the quality of fingerprint images. Hence, more study on the pre-processing phase can be considered to improve the outcome for the next phases (i.e. core-point detection, feature extraction). Thus, it should be possible to have robust features prior to recognizing the fingerprint image. However, such a feature extraction method must consider the hardware limitations on a mobile phone device.
