# Erratum to: Reference point detection for camera-based fingerprint image based on wavelet transformation

**DOI:** 10.1186/s12938-016-0141-x

**Published:** 2016-03-14

**Authors:** Mohammed S. Khalil

**Affiliations:** Faculty of Commerce and Economics, Sana’a University, Sana’a, Yemen; Center of Excellence Information Assurance, King Saud University, Riyadh, Saudi Arabia

## Erratum to: BioMedical Engineering OnLine (2015) 14:40 DOI 10.1186/s12938-015-0029-1

After publication of this article [1], we became aware that we had omitted to state that all individuals whose fingerprints were included gave their written consent for publication.

In addition, the following permission acknowledgements should have been included in the legends to the figures:

Figure 1: Reprinted from Weng et al. [2], Copyright 2011, with permission from Elsevier.

Figure 2: © 2009 IEEE. Reprinted, with permission, from Zhou et al. [3].

Figure 3: Reprinted with permission from Yang et al. [4].

Figure 18: Reprinted from Le and Van [5], Copyright 2012, with permission from Elsevier.
